# The Impact of Blood Pressure on Kidney Function in the Elderly: A Cross-Sectional Study

**DOI:** 10.1159/000355769

**Published:** 2014-04-09

**Authors:** Ya-Ping Zhang, Xiao-Cong Zuo, Zhi-Jun Huang, Ze-Min Kuang, Ming-Gen Lu, Dayue Darrel Duan, Hong Yuan

**Affiliations:** a Department of Cardiology, Central South University, Changsha, China 410013; b Center of Clinical Pharmacology, the Third Xiang-Ya Hospital, Central South University, Changsha, China 410013; c School of Community and Health Sciences, University of Nevada, School of Medicine, Reno, Nevada, USA 89557; d Laboratory of Cardiovascular Phenomics, the Department of Pharmacology, University of Nevada, School of Medicine, Reno, Nevada, USA 89557

**Keywords:** Hpertension, Hypotension, Obesity, Hyperlipidemia, Diabetes, Glomerular filtration rate, Rrenal insufficiency, Proteinuria

## Abstract

**Background/Aims:**

Intensive blood pressure (BP) target decreases blood perfusion of kidneys that attenuates the benefits of BP treatment in elderly hypertensive individuals. The optimal BP goal for renal function in the hypertensive elderly has been unclear. We investigated the impact of BP on renal function to define the appropriate BP target in the elderly.

**Methods:**

A total of 28,258 elderly subjects were categorized into normotensive (Norm), hypotensive (Hypo) and hypertensive (Hyper) groups according to BP levels. Systolic, diastolic and pulse BP (SBP, DBP and PBP) were further stratified by 10 mmHg. Blood urea nitrogen, serum creatinine, uric acid, glomerular filtration rate (GFR), renal insufficiency prevalence (RIP) and proteinuria prevalence (PP) were compared among different groups and BP strata. The RIP and PP in the elderly with obesity, hyperlipidemia or diabetes in Norm, Hypo and Hyper groups were evaluated.

**Results:**

GFR in Hypo and Hyper groups was significantly lower than that in Norm group. The RIP and PP was higher in Hypo and Hyper groups than that in the Norm group. Proteinuria became more prevalent when SBP was >140 mmHg or <90 mmHg. DBP>80mmHg increased PP while DBP<70 mmHg increased RIP. PBP>60 mmHg led to an increased RIP and PP. Obesity or hyperlipidemia only combined with hypertension caused a significantly increased RIP and PP. Diabetes independent of hypertension contributed to higher RIP and PP.

**Conclusions:**

The most beneficial BP target for kidney function in the elderly may be SBP of 90-140 mmHg and DBP of 70-80 mmHg. PBP <60 mmHg may be appropriate.

## Introduction

The prevalence of hypertension has been on the rise which causes a large global economic burden worldwide [1, 2]. Hypertension is a major independent risk factor for cardiovascular events, cerebrovascular events and renal disease [3-9]. Hypertension-induced kidney damage is associated with increased cardiovascular morbidity and mortality [10-13]. The coexistence of hypertension and obesity [14, 15], hyperlipidemia [16] or diabetes [17] results in worse renal dysfunction than isolated occurrence of either risk factor alone. Evidence from numerous clinical trials has demonstrated benefits of blood pressure (BP) control [18]. The target of 140/90mmHg that has been established from observational data remains fully justifiable. However, it is unclear whether the available results could be extrapolated to elderly patients. It is unknown whether the target of 140/90mmHg is most optimal goal to protect elderly renal function. It is debatable about the BP targets when hypertension in the elderly is complicated by coexist with obesity, hyperlipidemia or diabetes. The lower target limit of BP for hypertension treatment is still not determined. On the other hand, Intensive hypertension treatment probably causes hypotension in elderly patients which is also an independent predictor of cardiovascular disease and of all-cause mortality [19]. Usually hypotension including orthostatic and postprandial hypotension is neglected among elderly. In this study, we investigated the impact of BP on renal function to define the appropriate BP target in the elderly. We also evaluated how hypertension and hypotension combined with obesity, hyperlipidemia, or diabetes impacted renal function to highlight the importance of overall management in hypertensive elderly patients.

## Material and Methods

The study protocol was approved by the medical ethics committee of the Third Xiang-ya Hospital. All of the subjects enrolled in this study gave informed consent to participate in this study.

### Study Subjects

In this cross-sectional study, 31,941 Chinese people were consecutively recruited who participated in annual physical examination in Health Examination Center of the Third Xiang-Ya Hospital in Hunan Province between 2008 and 2010. Inclusion criteria were >60 year-old, male or female. Exclusion criteria included 1) no available data on BP value, glucose concentration, cholesterol level, blood urea nitrogen (BUN) level, uric acid (UA) level, serum creatinine (Scr) level, urine analysis, body weight, or height, and 2) history of primary renal diseases; and 3) renal artery stenosis, and 4) duplicate cases. A total of 28,258 subjects met with the inclusion and exclusion criteria and were eligible for inclusion in this study. Hypertension was defined according to the JNC 7 Report [18], and people who had previously received a diagnosis of hypertension were also considered to have hypertension. Hypotension was considered to be systolic blood pressure (SBP) less than 90mmHg or diastolic blood pressure (DBP) less than 60mmHg [20, 21]. The presence of obesity was defined by body mass index (BMI) of 28kg/m^2^ or more [18]. Hyperlipidemia was defined as serum concentration of total cholesterol (TC) of more than 5.7mmol/L, triglycerides of more than 1.7mmol/L [18], or patients who had previously received a diagnosis of hyperlipidemia and been being treated by statin drugs were also considered to have hyperlipidemia. Diabetes was diagnosed by fasting blood glucose of 7.0mmol/L or more, casual blood pressure of 11.1mmol/L or more [22] or patients had a previous diagnosis of diabetes. Renal insufficiency [23] was defined as estimated glomerular filtration rate (eGFR) < 60 ml/min/1.73m^2^.

### Measurements and Prediction Formulas

The examination was carried out during outpatient visit including an interview to determine the history of hypertension, diabetes and hyperlipidemia. After fasting overnight, BP was measured with an appropriately sized cuff and a mercury column sphygmomanometer in the sitting posture after a 10-min rest. Height and body weight were measured, and BMI was calculated as body weight divided by square of height. Venous blood sampling from all of the subjects was performed. Serum glucose, TC, high density lipoprotein (HDL) cholesterol, low density lipoprotein (LDL) cholesterol, triglycerides, blood urea nitrogen (BUN), Scr and uric acid (UA) rand Scr level were determined using standard laboratory methods. Qualitative analysis of urea protein was measured by sulfosalicylic acid method. GFR was estimated using the simplified Modification of Diet in Renal Disease (MDRD) and chronic kidney disease (CKD-EPI) equations. MDRD estimate of kidney function was calculated as 186×Scr^-1.154^×Age^-0.203^ (×0.742 if female). The CKD-EPI estimate of renal function was calculated as recommended: For women with a Scr ≤0.7, 144×(Scr/0.7)^-0.329^×0.993^age^; for women with a Scr>0.7, 144×(Scr/0.7)^-1.209^×0.993^age^; for men with a Scr ≤0.9, 141×(Scr/0.9)^-0.411^×0.993^age^; for men with a Scr >0.9, 141×(Scr/0.9)^-1.209^×0.993^age^.

### Statistical Analysis

Continuous variables were expressed as mean ± SD and categorical variables as percentage unless otherwise stated. The subjects were categorized into normotensive (Norm), hypotensive (Hypo) and hypertensive (Hyper) groups according to BP level. Each of Norm, Hypo and Hyper groups was further divided into control, obesity, hyperlipidemia and diabetes groups. One-way ANOVA was used to compare difference of means of continuous variables among three groups or more. Analysis of covariance was used to control for confounding factors. The significance of difference between two groups was determined by chi-square test for categorical variables and *t* test for continuous variables. Multiple linear regression was performed to analyze the relationship between blood pressure and clinical or laboratory parameters. Systolic, diastolic and pulse pressure (PBP) were stratified by 10mmHg. Binary logistic regression was used to explore the effect of different SBP, DBP and PBP strata on renal insufficiency and proteinuria. SBP stratum of 100-110 mmHg, DBP stratum of 70-80 mmHg and PBP stratum of 40-50 mmHg were considered as reference. Odds ratio (OR) and 95% confidence internal (CI) were calculated. The validity of the models was confirmed by conducting the likelihood-ratio test. *P* value under 0.05 was considered to be statistically significant. Analysis was performed in statistical software package SPSS16.0 for Windows.

## Result

### Summary of all study subjects

**Fig. 1:**
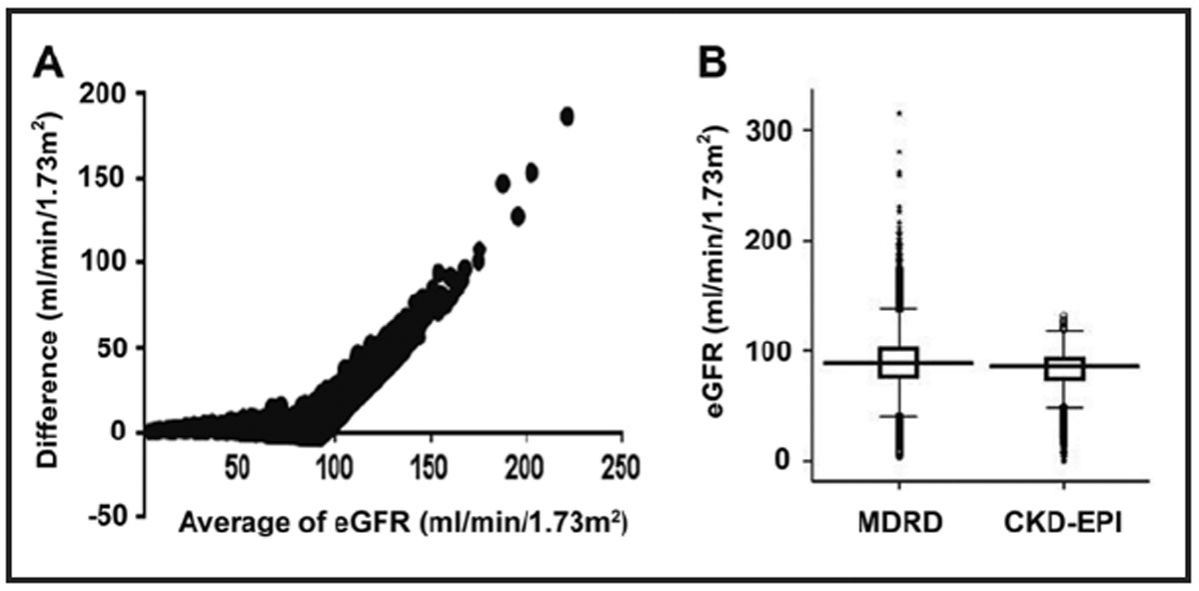
Comparison between MDRD formula and CKD-EPI equation in the elderly. A: Bland-Altman plot of MDRD and CDK-EPI eGFR; B: Boxchart of MDRD and CDK-EPI eGFR. ★ represents extreme values, ○ represents outliers.

Overall, 28,258 subjects with 66% male and at age of 69.4±6.2 years were studied. The prevalence of hypertension, hypotension, obesity, hyperlipidemia and diabetes were 68.2%, 4.4%, 10.4%, 41.2% and 47.6%, respectively. Males were more likely to have hypertension than females (69% vs 67.7%; *P*<0.0001) but females were more likely to have hypotension than males (6.2% vs 3.4%, *P*<0.0001). SBP was as high in women as in men (141±20.3 vs 141±19.3 mmHg; *P*=0.953) whereas women had lower DBP and larger PBP than men (76±11.7 vs 79±11.9 mmHg; 65±16.4 vs 62±15.6 mmHg; *P*<0.0001). Estimated GFR by MDRD formula was higher than that calculated by CKD-EPI equation (89.7±20.2 vs 82.6±14.1 ml/min/1.73m^2^; *P*<0.0001). There was a mean difference of 3.5ml/min/1.73m^2^ between MDRD and CKD-EPI eGFR when their average was less than 98ml/min/1.73m^2^ but the difference became larger and larger when their average value was more than 98ml/min/1.73m^2^. Many extreme high values of GFR could be easily obtained by MDRD equation (Figure 1).

### Effects of hypertension and hypotension on renal function

The general characteristics of study subjects within Norm, Hypo and Hyper categories are shown in Table 1. The relationship between blood pressure and parameters including age, sex, BMI, cholesterol, glucose, BUN, Scr, UA or GFR in hypotension and hypertension was analyzed by multiple linear regression as shown in Table 2. Increased SBP and decreased DBP separately contributed to development of hypertension and hypotension in elderly both of which led to larger PBP. Compared with normotensive subjects, hypertensive people were more likely to be older, male, have obesity, hyperlipidemia, diabetes and higher levels of BUN, Scr and UA. Hypotensive people were more likely to be older, female, and have lower levels of BMI, TC, Scr, UA but higher BNU and prevalence of diabetes. However both hypertensive and hypotensive subjects had lower GFR and higher prevalence of renal insufficiency. After adjustment for confounding factors (age, gender, BMI, cholesterol and glucose), both hypertension and hypotension groups still had decreased GFR (*P*<0.0001). Hypertensive group had 1.93-fold higher prevalence of renal insufficiency (95% CI 1.71 to 2.17; *P*<0.0001) and 2.24-fold higher prevalence of proteinuria (95% CI 2.05 to 2.45; *P*<0.0001) than normotensive group, while hypotensive group had 1.84-fold higher prevalence of renal insufficiency (95% CI 1.46 to 2.32; *P*<0.0001). But there was no significant difference in prevalence of proteinuria between hypotensive and normotensive groups (*P*=0.583). The multiple linear regression showed that 1) cholesterol and glucose levels were positively related to blood pressure only in hypertension group; 2) age was a negative factor for DBP; 3) BMI was positively correlated with blood pressure in both hypotension and hypertension groups; and 4) a decreased GFR was positively correlated to not only hypertension but also hypotension (Table 2).

### Effects of SBP in different strata on renal function

**Fig. 2:**
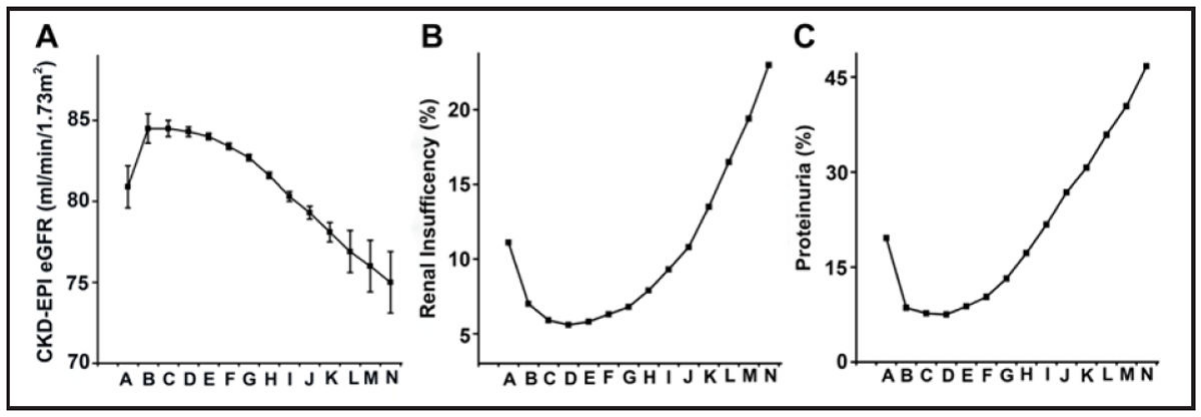
Effects of different SBP strata on GFR, renal insufficiency prevalence and proteinuria prevalence. GFR was expressed in mean±SE. A, B, C, D, E, F, G, H, I, J, K, L, M and N represents 80-89mmHg, 90-99mmHg, 100-109mmHg, 110-119mmHg, 120-129mmHg, 130-139mmHg, 140-149mmHg, 150-159mmHg, 160-169mmHg, 170-179mmHg, 180-189mmHg, 190-199mmHg, 200-209mmHg and 210-220mmHg stratum, respectively.

The GFR and prevalence of renal insufficiency and proteinuria in different SBP strata are presented in Figure 2. When SBP decreased from stratum 90-100mmHg to stratum 80-90mmHg, GFR was reduced by 3.6ml/min/1.73m^2^ (*P*=0.013) while BUN, Scr and UA respectively increased by 0.2mmol/L, 1.93μmol/L and 7.5μmol/L (all *P*>0.05). GFR decreased progressively and continuously with increase in SBP, approximately going down by 1.13ml/min/1.73m^2^ for every 10mmHg increase in SBP that occurs within the range of 140 to 230mmHg while BUN, Scr and UA increased by 0.08mmol/L, 1.54μmol/L and 1.73μmol/L for every 10mmHg increase in SBP. OR values of renal insufficiency and proteinuria in different SBP strata are shown in Table 3.

### Effects of DBP in different strata on renal function

**Fig. 3:**
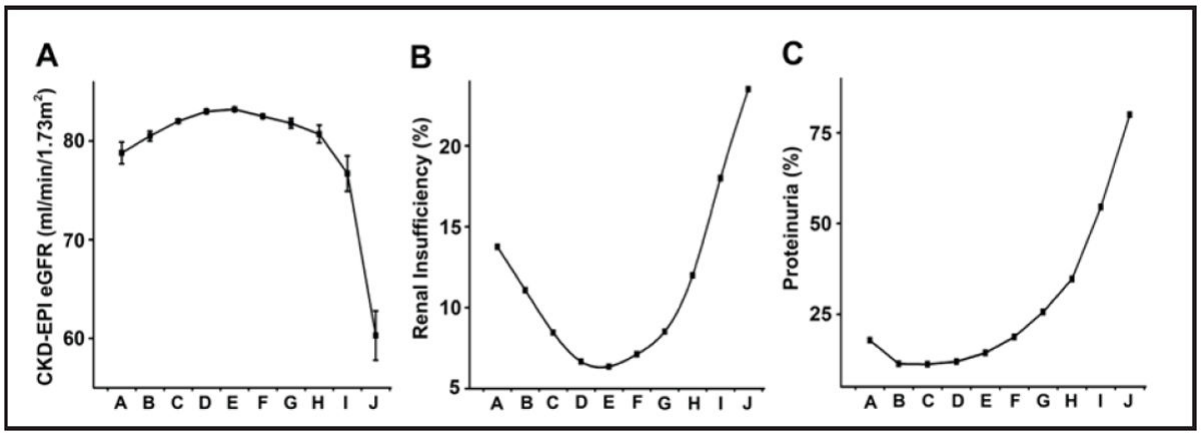
Effects of different DBP strata on GFR, renal insufficiency prevalence and proteinuria prevalence. GFR was expressed in mean±SE. A, B, C, D, E, F, G, H, I and J represents 40-49mmHg, 50-59mmHg, 60-69mmHg, 70-79mmHg, 80-89mmHg, 90-99mmHg, 100-109mmHg, 110-119mmHg, 120-129mmHg and 130-139mmHg stratum, respectively.

The GFR and prevalence of renal insufficiency and proteinuria in different DBP strata are presented in Figure 3. As DBP less than 70mmHg went down, GFR was reduced by 1.4ml/min/1.73m^2^ for every 10mmHg decrease while BUN, Scr and UA respectively increased by 0.1mmol/L, 0.63μmol/L and 1.33μmol/L. When DBP ranging from 90-120mmHg increased, GFR decreased by 1.93ml/min/1.73m^2^ for every 10mmHg change while BUN, Scr and UA increased 0.03mmol/L, 2.8μmol/L and 2.95μmol/L. When DBP was over 120mmHg, GFR obviously decreased and BUN Scr and UA quickly increased. OR values of renal insufficiency and proteinuria in different DBP strata are shown in Table 4.

### Effects of PBP in different strata on renal function

**Fig. 4:**
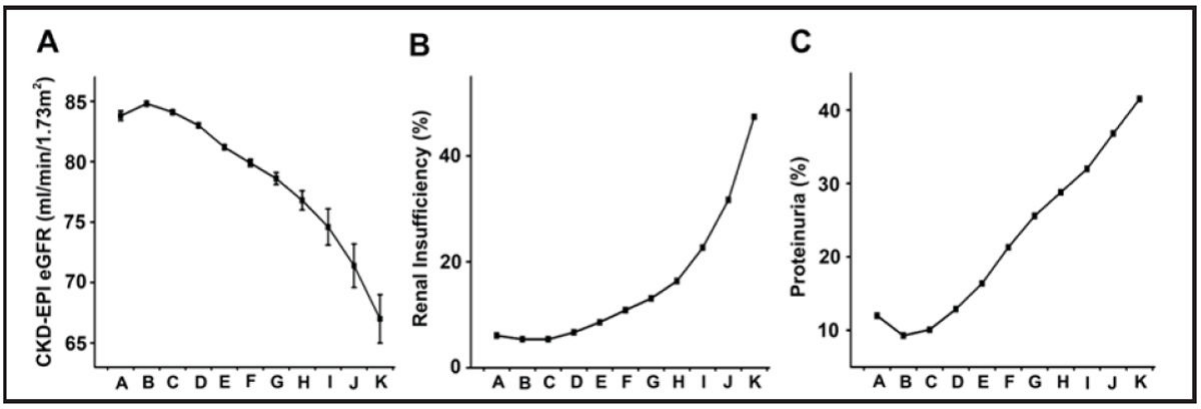
Effects of different PBP strata on GFR, renal insufficiency prevalence and proteinuria prevalence. GFR was expressed in mean±SE. A, B, C, D, E, F, G, H, I, J and K represents <40mmHg, 40-49mmHg, 50-59mmHg, 60-69mmHg, 70-79mmHg, 80-89mmHg, 90-99mmHg, 100-109mmHg, 110-119mmHg, 120-129mmHg and 130-139mmHg, respectively.

The GFR and prevalence of renal insufficiency and proteinuria in different PBP strata are presented in Figure 4. As PBP over 60mmHg went up, GFR was reduced by 2.29ml/min/1.73m^2^ for every 10mmHg increase while BUN, Scr and UA respectively increased by 0.16mmol/L, 2.27μmol/L and 5.18μmol/L. OR values of renal insufficiency and proteinuria in different PBP strata are shown in Table 5.

### Effects of hypertension and hypotension combined with obesity, hyperlipidemia or diabetes on renal function

**Fig. 5:**
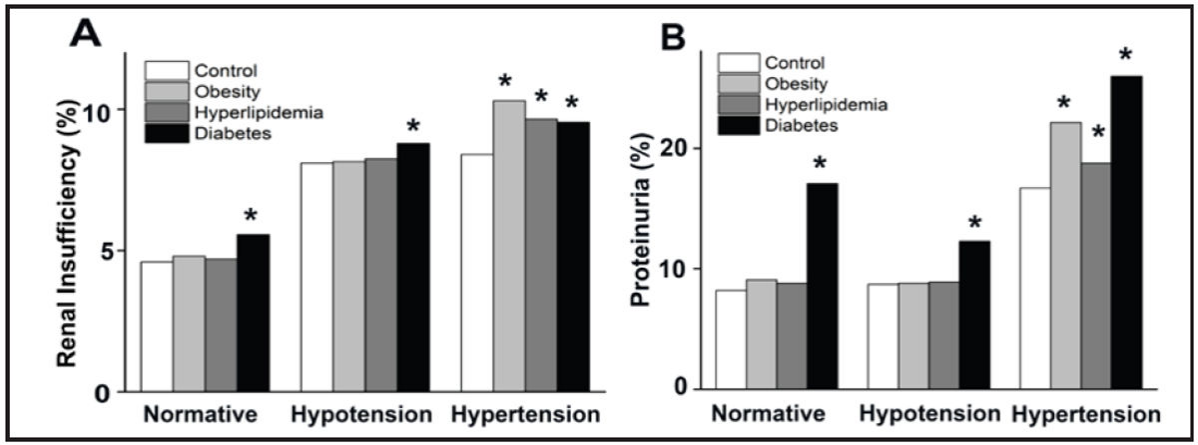
Effects of obesity, hyperlipidemia or diabetes on renal insufficiency and proteinuria in the elderly. Renal insufficiency rate and proteinuria rate of all the normotensive, hypotensive, hypertensive subjects were used as control. *P<0.05 vs control.

Renal insufficiency and proteinuria rate of the hypertensive and hypotensive elderly with obesity, hyperlipidemia or diabetes are presented in Figure 5. Diabetes significantly increased prevalence of renal insufficiency and proteinuria in all elderly. In hypotensive group, obesity and hyperlipidemia did not have effect on renal arterial perfusion and proteinuria but both of them could enhance damage to renal function in hypertensive group. There is no interaction on GFR between hypertension or hypotension and obesity, hyperlipidemia or diabetes (*P*>0.05).

## Discussion

In total, we studied 28,258 elderly subjects including hypertension, hypotension, obesity, hyperlipidemia and diabetes patients. Given the difficulties with accurate estimation of GFR and the limitations of calculating GFR by the MDRD formula, we used the new CKD-EPI equation. Although overall mean bias is smaller for the MDRD formula, the higher accuracy is reached with the CKD-EPI equation [24]. Compared with the CKD-EPI formula, the GFR values obtained by the MDRD method were higher and had larger variance, especially when GFR is ≥98ml/min/1.73m^2^.

The prevalence of hypertension increases markedly with age, such that approximately two thirds of people over 60 years of age have hypertension. This study showed the prevalence of hypertension was 68.2% among elderly in 2008 to 2010 which increased by 19% than that in 2002 [25]. It is clear that average SBP and DBP in men less than 60 years of age are higher than in age-matched women by 6-7 and 3-5mmHg [26, 27] however BP (particularly SBP) increased in women so that hypertension became more prevalent or at least as prevalent in women as men after 60 years of age [28]. Some authors reported gender was not associated with elderly hypertension. In our elderly-based study SBP had no difference between female and male but DBP in men was still higher by 3mmHg, suggesting SBP in women over age of 60 years increases quickly. Hypertension was still more prevalent in men than women. The pattern of blood pressure elevation changes with age. For most elderly, DBP starts to decline after age 60 although SBP increases steadily with age. PBP therefore widens with age. Isolated systolic hypertension predominates among elderly and it is present in 65% of all hypertensive elderly for which this study supplies strong evidence. Hypotension is usually understood as a physiological state, rather than a disease. There are very few reports about hypotension in the elderly. An orthostatic decline in blood pressure accompanies advanced age and sometimes is an inevitable adverse effect of some antihypertensive drugs. Orthostatic hypotension is an independent predictor of cardiovascular disease and of all-cause mortality [19]. It is a marker of poor prognosis in older patients. In this study, 4.4% of elderly suffered hypotension most of whom are female.

It is well known that hypertension could cause severe renal damage including decreased GFR, high prevalence of renal insufficiency and proteinuria [8, 9, 19]. The current cross-section study not only further confirmed these notions but also provided novel and important evidence suggesting that hypotension might also be related to renal dysfunction as a potential risk factor. In normotensive population of this study, the prevalence of renal insufficiency was 4.6%, similar to those performed in Spanish and Canadian population [29,30]. However patients with hypertension had 1.93-fold higher risk of renal insufficiency and 2.24-fold higher prevalence of proteinuria than normotensive elderly. In hypotensive elderly patients, the risk of renal insufficiency was 1.84-fold higher than normotensive population and, correspondingly, the prevalence of renal insufficiency significantly rose from 4.6% to 8.1% (*P*<0.0001); the prevalence of proteinuria also went up from 8.2% to 8.7% although the statistical analysis showed no significant difference between hypotensive and normotensive groups (P=0.583).

Data from randomized controlled trials suggests that treating hypertension in the elderly may substantially reduce the risk of cardiovascular disease and kidney damage [18]. However treatment remains a challenge because of aging-related changes. Therapy should be considered carefully in all aging hypertensive patients. At present the generally recommended blood pressure goal of 140/90 mmHg for hypertensive elderly patients is based on expert opinion. Target for BP management has not been based on observational data in the elderly. Elevated SBP is a very important risk factor for both cardiovascular and renal disease in the elderly [18, 31] and the evidence supporting a target SBP level <140 mmHg in slowing progression of kidney disease is strong. We found the elderly with SBP of 150-160 mmHg had 1.24-fold risk of renal insufficiency considering SBP ranging from 100 to 110 mmHg as reference and OR of renal insufficiency increased by 1.45 fold when SBP changed from 150-160 mmHg to 160-170mmHg We also found proteinuria was relevant to SBP. Proteinuria risk increased by 1.62 fold in the elderly with SBP stratum of 140-150mmHg. So this study supports the treatment target of SBP <140 mmHg in the elderly in order to protect renal function. But until now there has been limited data regarding the effect of lowering BP to <130 mmHg and benefit of intensive antihypertensive treatment. Our study did not show target of SBP <130 mmHg was more beneficial for elderly kidney than target of SBP <140 mmHg Given the limitation in quality and applicability of published data, ongoing randomized, multicenter Systolic Blood Pressure Interventional trial (SPRINT) is expected to provide the evidence needed to support standard vs aggressive hypertension control among the elderly. The trial is projected to run not until late 2018. One report showed DBP and PP had no significant association with a decline in kidney function in the elderly [32]. But the current study showed that DBP of 70-80 mmHg might be best beneficial for kidney function of the elderly. A decreased DBP to <70 mmHg was found to be related to renal insufficiency while an increased DBP to >80 mmHg was related to prevalence of proteinuria. However, further longitudinal and prospective studies are needed for a clear conclusion on whether there is indeed a causative relationship between hypotension and chronic kidney dysfunction or whether hypotension might be a potential risk factor of chronic renal disease. PBP is a predictor of adverse outcomes of chronic kidney disease and associated with decline in kidney function [33]. This study showed when PBP was over 60mmHg renal perfusion was significantly reduced and proteinuria was produced.

Diabetes increases prevalence of renal insufficiency and proteinuria among the elderly [34, 35]. It is questionable that whether the hypertensive elderly with diabetes need more aggressive goal of BP in preventing the incidence of diabetic kidney and slowing its progression [17, 36]. Some studies showed SBP under 130 or 120 mmHg in the diabetic elderly increased risk of total and cardiovascular disease (CVD) mortality and serious side effects [37, 38]. Based on a recent meta-analysis, it was concluded that, among patients with type 2 diabetes, the more aggressive target for SBP of <130 mmHg has to be balanced between the benefit of lowering stroke incidence, such as life-threatening event or hospitalization, and the lack of benefit for cardiac, renal and retinal outcomes [39-43]. Our study is consistent with those previous reports, revealing SBP of 130-140 mmHg does not increase prevalence of proteinuria (OR=0.69). The DBP stratum of 80-90mmHg mildly increases risk of proteinuria (OR=1.26, *P*=0.012). However the Irbesartan in Diabetic Nephropathy Trial (IDNT) showed progressive lowering of BP up to DBP of 85 mmHg protected against cardiovascular event but not renal endpoint [44, 45]. Therefore, cautions should be paid when applying the guideline to lower BP to 130/80 mmHg in patients with diabetic nephropathy universally. It is still not clear if elderly patients with hypertension confronted with obesity or hyperlipidemia would need different BP goal [46-48]. Our study revealed hypertension with obesity or hyperlipidemia led to worse kidney dysfunction than it alone in the elderly.

## Conclusion

Our findings suggest that while hypertension has a clear causative relationship with chronic renal damage as has been reported previously hypotension may also contribute to renal dysfunction in the elderly. And the most beneficial BP target for kidney function in the elderly may be SBP of 90-140 mmHg and DBP of 70-80 mmHg. PBP less than 60 mmHg may be appropriate. Only obesity or hyperlipidemia combined with hypertension causes kidney damage. They do not impact renal function alone. However, diabetes independent of hypertension leads to renal insufficiency and proteinuria. Diabetes confronted with hypertension enhances renal dysfunction. The hypertensive elderly patients with obesity hyperlipidemia or diabetes might need different BP target.

The strategy and intensity of treatment of BP in the elderly should be based on the patient’s individual CVD risk factors and possible comorbidity conditions rather than on aggressive BP goal recommended by global guideline alone. Although our study supplies important primary evidence about BP goal in the elderly further large prospective elderly-based study will be needed to confirm our observations.

## Figures and Tables

**Table 1:** Characteristics of subjects in normotensive, hypotensive and hypertensive groups

Variables	Groups			*P*
	Normotensive	Hypotensive	Hypertensive	
N	7745	1237	19276	
Age (yr)	68.2±5.9	71.2±7.1	69.8±6.2	<0.0001
Male (%)	65.9	51.6	66.3	<0.0001
BMI (kg/m^2^)	23.6±3	22.5±3	24.6±3.1	<0.0001
SBP (mmHg)	126±9.6	115±12	148±18.1	<0.0001
DBP (mmHg)	74±7.1	56±3.8	81±11.9	<0.0001
PBP (mmHg)	52±9.3	59±12.6	68±16.1	<0.0001
Cholesterol (mmol/L)	5±0.9	4.9±0.9	5±0.9	<0.01
Glucose (mmol/L)	5.3±1.4	5.3±1.3	5.6±1.5	<0.0001
BUN (mmol/L)	5.3±1.4	5.5±1.6	5.4±1.6	<0.0001
Scr (μmol/L))	73.8±18.5	72.8±20.4	77.8±28.3	<0.0001
UA (μmol/L))	311.6±82.8	302.4±86	329.7±89.2	<0.0001
CKD-EPI eGFR (ml/min/1.73m^2^)	85.1±12.6	82.4±14	81.6±14.5	<0.0001
Renal insufficiency (%)	4.6	8.1	8.4	<0.0001
Proteinuria (%)	8.2	8.7	16.7	<0.0001
Obesity (%)	7.1	3.7	12.2	<0.0001
Hyperlipidemia (%)	37.4	31.9	43.3	<0.0001
Diabetes (%)	13	14.9	19.6	<0.0001

BMI, body mass index; SBP, systolic blood pressure; DBP, diastolic blood pressure; PBP, pulse pressure; BUN, blood urea nitrogen; Scr, serum creatinine; UA, uric acid; CKD-EPI eGFR, estimated glomerular filtration rate by chronic Kidney Disease Epidemiology Collaboration formula

**Table 2:** Relationship between Blood Pressure and Characteristic Parameters in Hypotension and Hypertension by Multiple Linear Regression

Parameters	Hypotension			Hypertension		

	βfor SBP	βfor DBP	βfor PBP	βfor SBP	βfor DBP	βfor PBP
Age (yr)	0.37*	-0.19*	0.43*	0.02*	-0.08*	0.09*
Sex(M/F)	0.14*	-0.17*	0.19*	0.05*	-0.14*	0.15*
BMI (kg/m^2^)	0.28*	0.13*	0.24*	0.09*	0.08*	0.02*
Cholesterol (mmol/L)	0.05	0.53	0.04	0.05*	0.1*	0.03*
Glucose (mmol/L)	0.05	-0.05	0.06	0.07*	0.02*	0.09*
BUN (mmol/L)	-0.02	-0.02	-0.01	-0.01	-0.08*	0.06*
Scr (μmol/L))	-0.05	-0.16	-0.00	-0.01	-0.09*	0.08*
UA (μmol/L))	-0.02	-0.04	-0.01	0.02	-0.04*	0.02*
CKD-EPI eGFR (ml/min/1.73m^2^)	0.18	0.19*	0.07	-0.15*	0.11*	-0.17*

The βvalues represent linear regression coefficient. The βof sex means the change caused by switch from male to female. BMI, body mass index; SBP, systolic blood pressure; DBP, diastolic blood pressure; PBP, pulse pressure; BUN, blood urea nitrogen; Scr, serum creatinine; UA, uric acid; CKD-EPI eGFR, estimated glomerular filtration rate by chronic Kidney Disease Epidemiology Collaboration formula.

* P < 0.05

**Table 3:** Prevalence and odds ratio of renal insufficiency and proteinuria in different SBP strata

SBP strata (mmHg)	Renal Insufficiency		Proteinuria	

	Prevalence (%)	OR (95% CI)	Prevalence (%)	OR (95% CI)
100-109	5.9	1.0 (reference)	7.7	1.0 (reference)
80-89	11.1	1.2(0.67-2.07)	19.6	2.6(1.63-4.10)*
90-99	7.0	0.5(0.26-1.04)	8.6	0.9(0.52-1.51)
110-119	5.6	0.8(0.57-1.07)	7.5	0.9(0.65-1.14)
120-129	5.8	0.8(0.62-1.10)	8.8	1.0(0.79-1.32)
130-139	6.3	0.9(0.68-1.17)	10.3	1.2(0.95-1.56)
140-149	6.8	0.9(0.70-1.22)	13.2	1.6(1.27-2.07)*
150-159	7.9	1.2(0.94-1.64)	17.2	2.2(1.72-2.81)*
160-169	9.3	1.5(1.09-1.93)*	21.7	2.9(2.29-3.78)*
170-179	10.8	1.5(1.04-2.16)*	26.8	3.9(2.99-5.05)*
180-189	13.5	1.5(1.14-2.10)*	30.7	4.0(3.00-5.05)*
190-199	16.5	2.7(1.76-4.04)*	35.9	6.8(5.00-9.98)*
200-209	19.4	3.2(1.81-5.72)*	40.4	7.1(5.32-13.71)*
210-220	23.0	3.4(1.91-5.89)*	46.7	9.3(5.81-14.90)*

Prevalence is shown as percentage; OR is shown with 95% CI. Logistic regression was used to evaluate the ORs of different SBP strata with 100-109 mmHg stratum as reference. ORs, odds ratio; CI, confidence interval.

* *P* < 0.05

**Table 4:** Prevalence and odds ratio of renal insufficiency and proteinuria in different DBP strata

DBP strata (mmHg)	Renal Insufficiency		Proteinuria	

	Prevalence (%)	OR (95% CI)	Prevalence (%)	OR (95% CI)
70-79	6.7	1.0 (reference)	11.9	1.0 (reference)
40-49	13.8	2.2(1.46-3.40)*	17.8	1.8(1.23-2.58)*
50-59	11.1	1.7(1.42-2.14)*	11.3	0.9(0.78-1.15)
60-69	8.5	1.3(1.14-1.47)*	11.2	0.9(0.84-1.05)
80-89	6.4	1.0(0.84-1.07)	14.3	1.2(1.13-1.35)*
90-99	7.1	1.2(1.01-1.36)	18.7	1.7(1.54-1.91)*
100-109	8.5	1.2(0.75-2.00)	25.6	2.6(2.17-3.00)*
110-119	12.0	1.3(0.98-1.60)	34.7	3.6(2.70-4.82)*
120-129	18.0	3.5(1.54-7.65)*	54.5	8.9(4.47-17.71)*
130-140	23.5	3.6(1.65-7.89)*	80.0	12.3(3.31-26.54)*

Prevalence is shown as percentage; OR is shown with 95% CI. Logistic regression was used to evaluate the ORs of different SBP strata with 70-79 mmHg stratum as reference. OR, odds, ratio; CI, confidence interval.

* *P* < 0.05.

**Table 5:** Prevalence and odds ratio of renal insufficiency and proteinuria in different PBP strata

PBP strata (mmHg)	Renal Insufficiency		Proteinuria	

	Prevalence (%)	OR (95% CI)	Prevalence (%)	OR (95% CI)
40-49	5.4	1.0 (reference)	9.3	1.0 (reference)
<40	6.1	1.0(0.73-1.31)	12.0	1.2(0.96-1.49)
50-59	5.4	1.0(0.83-1.17)	10.1	1.1(0.96-1.25)
60-69	6.7	1.3(1.06-1.48)*	12.9	1.4(1.26-1.64)*
70-79	8.6	1.7(1.39-1.96)*	16.4	1.9(1.67-2.17)*
80-89	10.9	2.1(1.77-2.56)*	21.3	2.6(2.28-3.04)*
90-99	12.1	2.4(1.92-2.98)*	25.6	3.5(2.96-4.14)*
100-109	13.4	2.7(1.66-4.34)*	28.8	3.9(3.33-5.22)*
110-119	17.7	3.2(2.44-4.30)*	32.0	4.2(2.72-5.65)*
120-129	30.7	9.7(5.07-18.45)*	36.8	5.7(3.66-12.91)*
130-140	47.4	15.7(6.30-38.96)*	41.5	6.9(2.21-14.46)*

Prevalence is shown as percentage; OR is shown with 95% CI. Logistic regression was used to evaluate the ORs of different SBP strata with 40-49 mmHg stratum as reference. OR, odds ratio; CI, confidence interval.

* *P* < 0.05.
